# Dermoscopy practice guidelines for use in telemedicine

**DOI:** 10.1038/s41746-022-00587-9

**Published:** 2022-04-27

**Authors:** Linda Camaj Deda, Rebecca H. Goldberg, Taylor A. Jamerson, Ivy Lee, Trilokraj Tejasvi

**Affiliations:** 1 University of Michigan Medical School, Ann Arbor, MI USA; 2 Pasadena Premier Dermatology, Pasadena, CA USA; 3 Department of Dermatology, University of Michigan, Ann Arbor, MI USA; 4 Ann Arbor Veteran Affairs, Ann Arbor, MI USA

**Keywords:** Health services, Physical examination, Education

## Abstract

Teledermoscopy, or the utilization of dermatoscopic images in telemedicine, can help diagnose dermatologic disease remotely, triage lesions of concern (i.e., determine whether in-person consultation with a dermatologist is necessary, biopsy, or reassure the patient), and monitor dermatologic lesions over time. Handheld dermatoscopes, a magnifying apparatus, have become a commonly utilized tool for providers in many healthcare settings and professions and allows users to view microstructures of the epidermis and dermis. This Dermoscopy Practice Guideline reflects current knowledge in the field of telemedicine to demonstrate the correct capture, usage, and incorporation of dermoscopic images into everyday practice.

## Introduction

Teledermatology involves a remote consultation for diagnostic and/or therapeutic advice. Teledermatology can occur by real-time video consultation or store-and-forward (SAF) images^1^. SAF images are prepared by general practitioners/patients or other specialists and sent to a dermatologist via secure portal, who makes recommendations for diagnosis and/or treatment after image examination^1^. Teledermoscopy is defined as the use of teledermatology to transmit dermoscopic images for remote consultation^1^.

Dermoscopy is an essential diagnostic tool for dermatologists to visualize epidermal structures, pigment patterns, and vascular patterns to aid examination of lesions and clinical decision-making. Access to dermoscopy images in addition to conventional telemedicine photographs is shown to significantly increase dermatologist diagnostic confidence^2^. Furthermore, addition of dermoscopic images improves efficacy and cost-effectiveness when used for skin cancer screening^3,4^. However, appropriate dermoscopy training is vital to proper and consistent use. User expertise and training increases diagnostic accuracy, whereas lack of training can pose major barriers to providers^5,6^. Teledermatologic modalities of care dramatically increased during the recent SARS-CoV-2 pandemic, yet there were increased numbers of missed melanomas and non-melanoma skin cancers, further necessitating proper dermoscopic provider competency in teledermatology visits^7,8^.

Additionally, as use of dermoscopy increases in fields outside of dermatology, there is need for proper dermatoscopic education in the primary care setting. Dermatoscopes in the primary care setting can inform decisions for biopsy and timely referral to dermatology, improve diagnostic accuracy of pigmented lesions, and facilitate earlier detection of melanoma and basal cell carcinoma^9–20^. Marwaha et al. showed that there is up to 9% greater probability of cancer detection with use of dermatoscopes compared with direct referral^21^. When compared to clinical photographs alone, use of dermatoscopes by well-trained primary care providers can also improve access to dermatologic care for underserved populations, reduce wait times, allow for continuous monitoring of lesions over time, and improve detection of suspicious lesions^22–25^.

While diagnostic accuracy in teledermoscopy is improved with high-quality images, up to 36% of dermoscopic images obtained by general practitioners during everyday practice were of poor quality^26^. Dermoscopy adds additional value in primary care when used by an expert or trained user^27–30^. A sequential intervention trial showed that use of dermoscopy by trained primary care providers improved sensitivity in diagnosis of melanoma and reduced benign-to-malignant excision ratio by assessing lesion stability^31,32^. A study of thirty-four healthcare practitioners demonstrated that use of mobile dermatoscopes in their practice was helpful for lesion monitoring, but reported technical tissues (33%) and uncertainty to advocate teledermoscopy for direct-to-consumer use (36%)^29^.

Additional challenges present as clinicians incorporate teledermoscopy along with gross lesion imaging into daily practice. If a physician does not recognize structures or incorrectly interprets them, this decreases diagnostic accuracy^33^. Diagnostic accuracy can also be further compromised if lesions are diagnosed without clinical context and a physical exam (i.e., palpation, stretch of skin, tenderness)^34^. Baseline and ongoing education for practitioners and proper dermatoscopes with accessories should be available.

Further limitations of teledermoscopy include safety and security concerns regarding data collection and storage, technical challenges in obtaining dermoscopy photos on genitalia and other anatomical locations, high cost of dermatoscopes (although this cost is decreasing as consumer-friendly mobile dermatoscopes are further developed), medico-legal concerns, reimbursement, and use of teledermoscopy by patients, which poses a new set of challenges related to user competency. Technical challenges associated with teledermoscopy (image orientation, resolution, scale, lighting, focus, color) are addressed in these guidelines.

Formal guidelines and requirements for acquiring dermoscopy images is crucial in ensuring standardized high-quality images for routine use in primary care settings. Currently, there are no guidelines or protocols for use of dermoscopy in telemedicine. Guidelines presented herein incorporate current recommendations with the goal of informing proper and standardized dermoscopic utilization in telemedicine to ensure high-quality images. We will describe guidelines for physical environment, patient evaluation and examination, follow-up care and coordination, devices, and equipment, including mobile device use, image capture and quality, storage, and requirements for asynchronous imaging. These guidelines will not cover interpretation of dermoscopy images, and it will not discuss in detail recommendations specific to patient-acquired mobile teledermoscopy, which can be found in Koh et al. 2021^35^.

The dermoscopy guidelines apply to individual practitioners, hospitals, practices, and any other healthcare teams who evaluate cutaneous lesions and rashes. Those who utilize these guidelines should comply with professional protocols set by his/her area of expertise on the diagnosis and treatment of skin lesions. These practical guidelines are for use within the United States (U.S.). Interactions, where either party resides outside of the United States, should consider local guidelines over the guidelines presented here, in accordance with the rules of prevailing jurisdictions.

The purpose of these guidelines is to provide practitioners a more comprehensive protocol for the use of dermatoscopes in telemedicine. The following expert-developed guidelines on the proper use of dermoscopy will help establish a standard of care to assist medical providers of diverse professions in the proper capture and storage of dermoscopic images for use in primary care, nursing, and more. These guidelines, protocols, and recommendations have been iteratively reviewed and consensus was reached by a panel of teledermatology experts of the American Telemedicine Association (ATA). Guidelines do not guarantee diagnostic accuracy, nor should they be followed in certain situations such as emergencies.

Refer to ATA Practice Guidelines for Teledermatology clinical practice guidelines, informed consent, physical environment, patient evaluation and examination, follow-up care and coordination, and documentation^36^.

## Results

To ensure clinical and technical benefit, teledermoscopy should follow a standardized approach and guidelines set forth on dermatoscope devices, image orientation, resolution, scale, measurement, focus, depth of field, color, and field of view.

### Devices

Dermatoscopes are available as independent devices, cellular device attachments, connections to a digital camera via coupling adaptors or direct lens attachments, and as a total dermoscopy system (pre-mounted dermoscopic lens on a high-quality camera linked directly to a computer). Use of digital cameras can prove beneficial to capture both regional, macroscopic, and dermoscopic images. Some digital cameras will require manual transfer of imaging, while those such as visioMed MicroDERM have wireless transfer^22^. A comprehensive list of devices can be found in Supplemental Tables 1 and 2.

### Image orientation

**Fig. 1:**
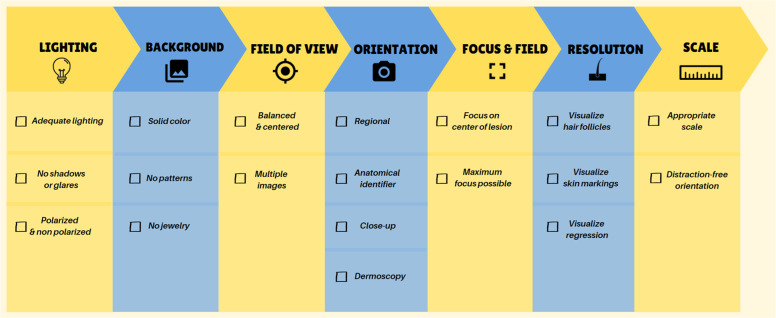
Guidelines for acquisition of clinical images. Follow this checklist to ensure proper lighting, background, field of view, orientation, focus, field, resolution, and scale when taking clinical images for store-and-forward utilization.

Image orientation shall be consistent to ensure accurate comparison over time. Regional images should have cephalic orientation, with the patient’s head toward the superior aspect of the image frame^32^. Vertical or horizontal orientation can be selected by discretion of the individual using the device and shall remain consistent for all follow-up images captured, including regional, close-up, and dermoscopic images. Regional images should include appropriate anatomical sites (i.e., a joint), to help orient and identify location of the lesion^32^. Orientation of dermoscopic images should be consistent with the corresponding gross image^25^. Finnane et al. and McKoy et al. provide additional complementary views and suggested image orientation based on body region; see Fig. 1 and Supplemental Fig. 2^32,37^.

### Resolution

Resolution is defined as the number of pixels in an image^32,38^. A device is considered to have acceptable resolution if hair follicles are visualized in regional images and skin markings are visualized in close-up images^32^. Dermoscopic images are considered to have appropriate resolution and magnification if dots and regression structures can be visualized^25,32,39^. These criteria will generally require a file size of at least 200KB^32^.

### Scale and measurement

A diameter scale shall be used to consistently measure and monitor lesion dimensions and morphology over time^25,32,39^. The scale should not obscure when used in close-up images^28^. Digital scales incorporated into the device should be used over physical scales to avoid inaccuracies associated with physical scales (i.e., poor placement of scale and/or obscuring of surrounding skin or lesions)^32,40^.

Software measurement tools can automatically generate a distance measurement. However, accurate measurement relies on the area of interest being aligned precisely parallel to the device sensor and the scale placed in the same orientation of the camera, measuring the longest diameter of the lesion. Independent of physical or digit scale utilization, the scale shall be placed in the same orientation as the dermatoscope^32^.

### Lighting

Selection of non-polarized light versus polarized light shall be left to the discretion of the individual using the device, guided by lesion characteristics^32^. However, polarized and nonpolarized dermoscopy can provide complementary information and it is generally recommended to capture at least one dermoscopic image with polarized light^25,32,39,41,42^.

Non-polarized light shall be used to visualize superficial structures and has higher specificity in diagnosing epidermal structures such as milia cysts, comedo-like openings in seborrheic keratoses, and blue-white veil associated with orthokeratosis^42–44^. Application of a gel or liquid such as ultrasound gel or alcohol-based hand sanitizer should be used to provide an interface between the skin surface and the device.

Polarized light eliminates superficial glare and should be used to visualize deep structures such as vasculature and collagen, and aid in identification of some malignant neoplasms^42–45^. Devices equipped with polarized-light do not allow for proper visualization of superficial structures, but can serve as an important diagnostic tool in assessing seborrheic keratoses^45^. Some dermatoscopes are capable of switching between non-polarized and polarized light and will blink if a lesion is visible in one mode and not in the other^46^.

### Focus/Depth of field

Depth of field defines the distance between the nearest and farthest objects that are in sharp focus in front of or behind the point of interest that the camera is focused on. This is influenced by focal length, distance to the lesion, and aperture. The center of the area of interest should be in the center of the frame^32,38^. The camera shall be positioned perpendicular (90 degrees) to the skin surface using a lens with a deep depth of field, allowing for maximum area of the image to be in focus^47^. Auto-focus can be used, or the user can first depress the shutter half-way to focus, then adjust the camera to the center of the image, and finally depressing the shutter button to capture the image. Smartphone cameras may have an autofocus built in, but to optimize the photo it is helpful to manually focus. When utilizing an iPhone camera, the image can be focused by using a finger to tap the lesion of interest on the screen. A yellow box will appear indicating that the focus has been set. On an Android device, images can be focused by activating manual or pro mode in camera settings, activating the focus “MF” (manual focus) icon, and utilizing a slider to focus manually.

### Color

Colors visualized by dermoscopy include yellow, brown, black, red, blue, gray, and white. The color of images taken over time shall be comparable to diagnose and monitor skin lesions correctly^32^. Based on recent ATA guidelines, an image color resolution of 24 bits is recommended for teledermatology applications^36^. Per manufacturer instruction, equipment shall be calibrated to prevent variability in color calibration and white balance between time points^32^. Standardization of color calibration for clinical or dermoscopic images has not been recommended to date^48^. We suggest utilization of Digital Imaging and Communications in Medicine (DICOM) for image transmission, processing, and storage given recent efforts being made by this platform to include metadata needed to derive standard colorimetry in medical imaging^49^.

### Field of view

The lesion or area of interest shall be centered when positioning the device^32^. Close up images of rashes should include 25:75% ratio of normal looking skin to rash^22^. Multiple images shall be captured if the longest axis of the lesion or focal area of interest is larger than the field of view captured by the device. All edges of the lesion shall be visualized^32^. Images should be taken with a device less than 2 inches from the skin in non-contact mode or touching the skin after application of alcohol wipe to both skin and device in contact mode to improve luminance^36^. Dermoscopic images should be captured using the same orientation as the corresponding close-up^39^.

### Quality

Providers obtaining dermoscopic images should be trained and technically competent dermatoscope utilization and have access to high-quality equipment. All clinicians should also have evidence of up-to-date nationally-accredited Continuing Professional Education (CPE) specific to dermoscopy and the clinical management of pigmented lesions in the five-year revalidation cycle^50^. Providers shall use a continuous quality improvement program, including a clinical oversight process. The quality improvement program includes:

- Technical or administrative failures.
- Appropriateness of virtual encounter.
- Patient and/or provider satisfaction.
- Patient outcomes.
- Pathology or imaging results.
- Recommendations for follow-up.
- Follow-up feedback on quality of images to the imager.

Additionally, for patients with pigmented lesions, dermoscopic images are an essential supplement for any teledermatology referral that is used to replace face-to-face consultation^50^. Providers and organizations shall uphold regular maintenance and testing of devices to ensure proper functioning of equipment and connectivity. A system-wide firewall and antivirus software shall be kept up-to-date.

### Store and forward consultation

**Fig. 2:**
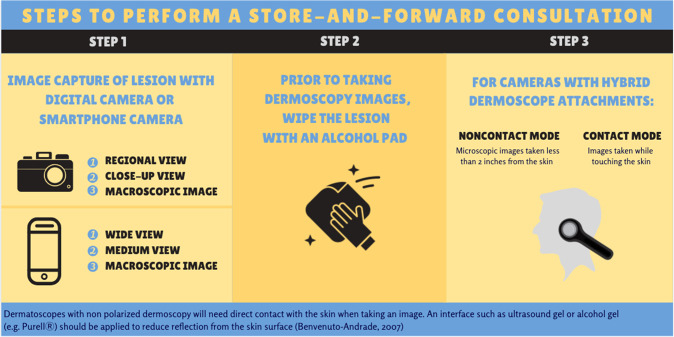
Store-and-forward consultation steps. Capture gross image of lesion. Wipe lesion with alcohol pad before taking dermoscopy photos.

Steps to perform a SAF consultation as described by Walocko and Tejasvi^17^. Please refer to Fig. 2 for steps to perform an SAF consultation and ATA guidelines for instructions for taking these images^36^.

### Teledermatologists

Consultant dermatologists should have experience and/or training in dermoscopic interpretation, including an understanding of limitations as well as appropriate selection of patients suitable for teledermatology. Half of teledermatologists have subspeciality in dermoscopy, and 33% subspecialize in skin cancer^2^. In order to minimize risk of luminance decay, the dermatologist should review images on a display less than five years old. Decreasing ambient lighting and/or increasing the brightness of the display can minimize reflection. Furthermore, it can be helpful to use software that permits image rotation, panning, and zooming^25^.

### Models of care

Teledermatology proves a valuable asset for diagnosis and management of dermatologic disease for underserved patients, including those in rural areas, medicaid populations, and the elderly. This being said, inherent reliance on and unequal access to internet connectivity and advanced devices could serve to worsen disparities. Further research is warranted in order to delineate the most effective ways to provide teledermatology for populations without access to in-person care, including the development of guidelines for non-physicians or non-providers collecting teledermoscopic images from remote locations^51,52^.

## Discussion

### Triage

Dermoscopy remains two-dimensional when viewing the images virtually or in person, which makes this an excellent tool for any teledermatology triage models. Patterns to differentiate benign from malignant have been very well described and so is the features for inflammatory dermatoses. For example, a pigmented lesion resembling seborrheic keratosis clinically could show concerning features on dermoscopy warranting a biopsy, Thus, the triage model could help in early detection of a malignant lesion. Similarly, if the lesion demonstrates dermoscopy patterns consistent with seborrheic keratosis, then reassurance is provided hence avoiding an unnecessary health visit. Inclusion of dermoscopy images may help discern, rashes, but evidence in literature is still evolving. Overall, utilizing dermoscopy in telemedicine for triage purposes improves access to expert consultation and dermatologic care when warranted.

### Caveats

Teledermatology, including teledermoscopy, should be used only in the appropriate clinical context when the provider is fully comfortable deferring in-person evaluation. For example, literature is limited for dermoscopy utility in rashes, though dermoscopic images in combination with clinical images have been found to improve the tele-diagnostic accuracy of pityriasis rosea and discoid lupus erythematosus compared to clinical images alone^53^. Providers must take a conservative approach, for there are inherent risks involved, including inability to perform total body skin exams, biases towards focusing solely on the lesion of interest, and ultimately harm to patients with possible legal repercussions for missed or misdiagnosed lesions^17^.

Dermosopy has demonstrated efficacy in improving the accuracy of detecting nonpigmented skin cancers compared to the unaided eye and assists with selection of appropriate management^54^. It is also increasingly utilized for monitoring and follow-up for skin cancers, as well as triage and follow-up of lesions which are changing, concerning, or clinically different from other lesions. For pigmented lesions, a full skin examination is useful to determine if the dermoscopic lesion of interest appears different from others on a given individual. Dermoscopy is an especially useful tool for patients with a history of melanoma, increased risk for melanoma, or presence of many atypical nevi.

However, it is unclear if dermoscopy is helpful in assessing melanomas smaller than 6 mm^33,55,56^. Certain lesions, such as Spitzoid proliferations and atypical melanocytic nevi, may have challenging dermoscopy features rendering this tool less useful^57^. Dermoscopy may be less appropriate or useful if there are technical challenges in obtaining a quality image in areas such as the genitalia and other anatomic sites such as the inside of the external ear, medial canthi, and nares. Hypo- and non-pigmented lesions also pose a challenge since identification of structural elements is more difficult. For pigmented nail lesions, dermoscopy can be a useful adjunct for recognizing patterns consistent with benign (eg, melanonychia) or malignant (eg, melanoma) entities. However dermoscopy of the nail should not replace microscopic examination which is necessary to exclude malignancy. Finally, certain lesional characteristics are best visualized under particular types of light, so clinicians must be aware of benefits and limitations of light sources^32^.

### Implications and call to action

Limited access to dermatology is a growing problem which is mitigated by implementation of teledermatology as an alternative to face-to-face visits. Incorporating teledermoscopy in primary care offers many benefits, including improved internet-based skin cancer screening, timely referral to dermatology, improved diagnostic accuracy of pigmented lesions, earlier detection of melanoma and basal cell carcinoma, remote evaluation for underserved populations, reduced wait times, cost effectiveness, reduced percentage of excisions, decreased malignant/benign excision ratio, post-biopsy review, educational opportunities, and continuous monitoring of lesions over time^3,22,24,27,44,58–69^. Given the multitude of benefits discussed, we recommend adoption of teledermoscopy with integration of these guidelines for appropriate image capture and assessment.

However, these benefits are only realized with proper user training, expertise, implementation, and workflow. Teledermoscopy is becoming more common in primary care. This set of guidelines and proposed framework directs clinicians on proper use to increase confidence, limit user errors, decrease under and misdiagnoses, and improve patient satisfaction with teledermoscopy. We recommend the development of and use of validated diagnostic criteria and characteristics when examining dermoscopic images of melanocytic and non-melanocytic lesions, and for determining whether skin lesions are suspicious. Although not discussed here, incorporation of computerized analyzing instruments or artificial intelligence (AI) can aid pattern recognition and facilitate dermoscopy use among primary care providers and dermatologists. These practice guidelines serve as a foundation for high-quality image capture and utilization in telemedicine and maximize the potential of telemedicine and artificial intelligence to benefit all patients.

Digital camera use, privacy, administration guidelines, security, licensing and credentialing, and liability should follow ATA guidelines.

## Methods

### Scope

These guidelines offer recommendations in consultation with experts in the field of teledermatology to delineate the proper use of teledermoscopy for all types of medical providers.

### Data acquisition

**Fig. 3:**
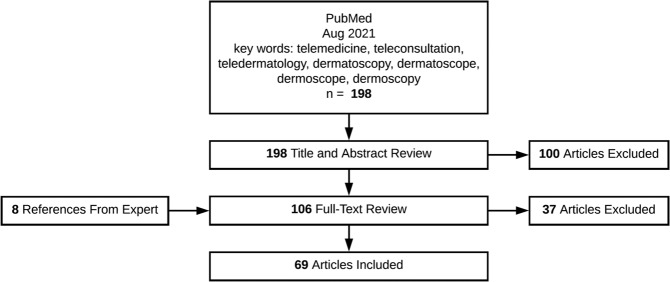
The Decision Tree of literature search. Literature search, review, and article inclusions.

We conducted a PubMed literature search with keywords: teleconsultation, teledermatology, telemedicine, dermatoscope, dermoscope, dermoscopy, and dermatoscopy. After screening abstracts for relevance, those focusing on teledermoscopy background information, indications, types, and recommendations for proper utilization were included, for a total of 69 articles excluding duplicates. Exclusion criteria include articles that were not written in English, those that were abstracts only, and those that focused on topics other than our topic of interest, such as telemedicine, artificial intelligence, machine-learning, reflectance confocal microscopy, and patient-utilized teledermatology applications. See Fig. 3 for literature search, review, and article inclusions. A comprehensive list of equipment for teledermoscopy, including independent devices, those customized for smartphone attachment, and digital camera dermoscopy were adapted from Teledermoscopy for Teledermatology by Singh et al. 2016 along with independent review of manufacturers websites^22^. For image orientation, guidelines for regional images were summarized from the existing literature on teledermatology and applied for consistent image capture via dermoscopy. Recommendations regarding resolution, scale, measurement, lighting, focus, depth of field, color calibration, and field of view are summarized and adapted from The International Skin Imaging Collaboration (ISIC), which includes 9 image criteria.

### Final review

The final product was reviewed by dermoscopy and telemedicine experts from the ATA Teledermatology Special Interest Group, including iterative revision to incorporate feedback and commentary.

### Reporting summary

Further information on research design is available in the Nature Research Reporting Summary linked to this article.

## Supplementary information

Supplementary Information

Reporting Summary
