# Supplementary material for: Dermoscopy practice guidelines for use in telemedicine
Source: NPJ Digit Med. 2022 Apr 27;5:55. doi: 10.1038/s41746-022-00587-9 (PMC9046409; doi:10.1038/s41746-022-00587-9)
Supplement: Supplementary file 1 — Supplementary Information [file 41746_2022_587_MOESM1_ESM.pdf]

Supplemental Table 1: Comprehensive list of dermatoscopes.

<table>
<tr><th colspan="7">Dermatoscopes</th></tr>
<tr><td colspan="7">Customized for Smartphone Attachment</td></tr>
<tr><td colspan="2">3Gen: DermLite</td><td colspan="2">Heine Optotechnik</td><td>Canfield Scientific</td><td>FotoFinder</td><td>MoleScope</td></tr>
<tr><td colspan="2">DL1
DL1 Basic
DL HÜD
Foto X
HandyScope
(DermLite/FotoFinder)</td><td colspan="2">iC1</td><td>VEOS DS3</td><td>Handyscope Classic

HandyScope (Dermlite/Foto Finder)</td><td>MoleScope</td></tr>
<tr><td colspan="7">Independent Devices</td></tr>
<tr><td>3Gen: DermLite</td><td>Heine Optotechnik</td><td>Canfield Scientific</td><td>DermoScan</td><td>Luxamed</td><td>Reister</td><td>KaWe</td></tr>
<tr><td>DL100
DL1
DL200
DL200 Hybrid
DL200 HR
DL3
DL3N
DL4
DL4W
DL GL
DL Carbon
DLII Pro
DERMLITE FOTO X & NAILIO
Lumio®
Lumio® UV
Lumio® S</td><td>DELTA 20T
DELTA 30
DELTAone
Mini3000
Mini3000 LED
NC2</td><td>microDERM Luminis (VISIOMED)

MicroDERM Optima (VISIOMED)

MicroDERM D200EVO (VISIOMED)

VEOS HD2</td><td>DermoGenius 3

DermoGenius Ultra

Illuco IDS-1100

Illuco IDS-3100 Magnum+</td><td>Luxascope 3.7V
Luxascope 2.5V</td><td>Ri-Derma 3.5V</td><td>Piccolight D
Eurolight</td></tr>
</table>

Supplemental Table 2: Comprehensive list of digital camera dermoscopy.

**Digital Camera Dermoscopy**					
3Gen: DermLite	*Caliber I.D.*	*Canfield Scientific*	*Derma Medical*	*Fotofinder*	*Visiomed*
DL Cam<br>DL Foto X<br>DL Foto X Plus<br>DL Foto II Pro<br>DL Foto II Pro Plus	VivaCam	VEOS SLR	MoleMax HD<br>MoleMax HD Pro<br>MoleMax HD Modular<br>MoleMax HD AMTBM<br>MoleMax 360<br>DermDOC HR<br>SkinDOC<br>PhotoMax PRO DLF IIicro	ATBM Master<br>Vexia<br>Medicam 1000<br>Leviacam	microDERM D120<br>microDERM E30<br>microDERM F80

Supplemental Figure 1: Example of low-quality versus high-quality image acquisition. Images should be cephalically oriented with consistent non-variable position of the lesion of interest for clinical and dermoscopic images.

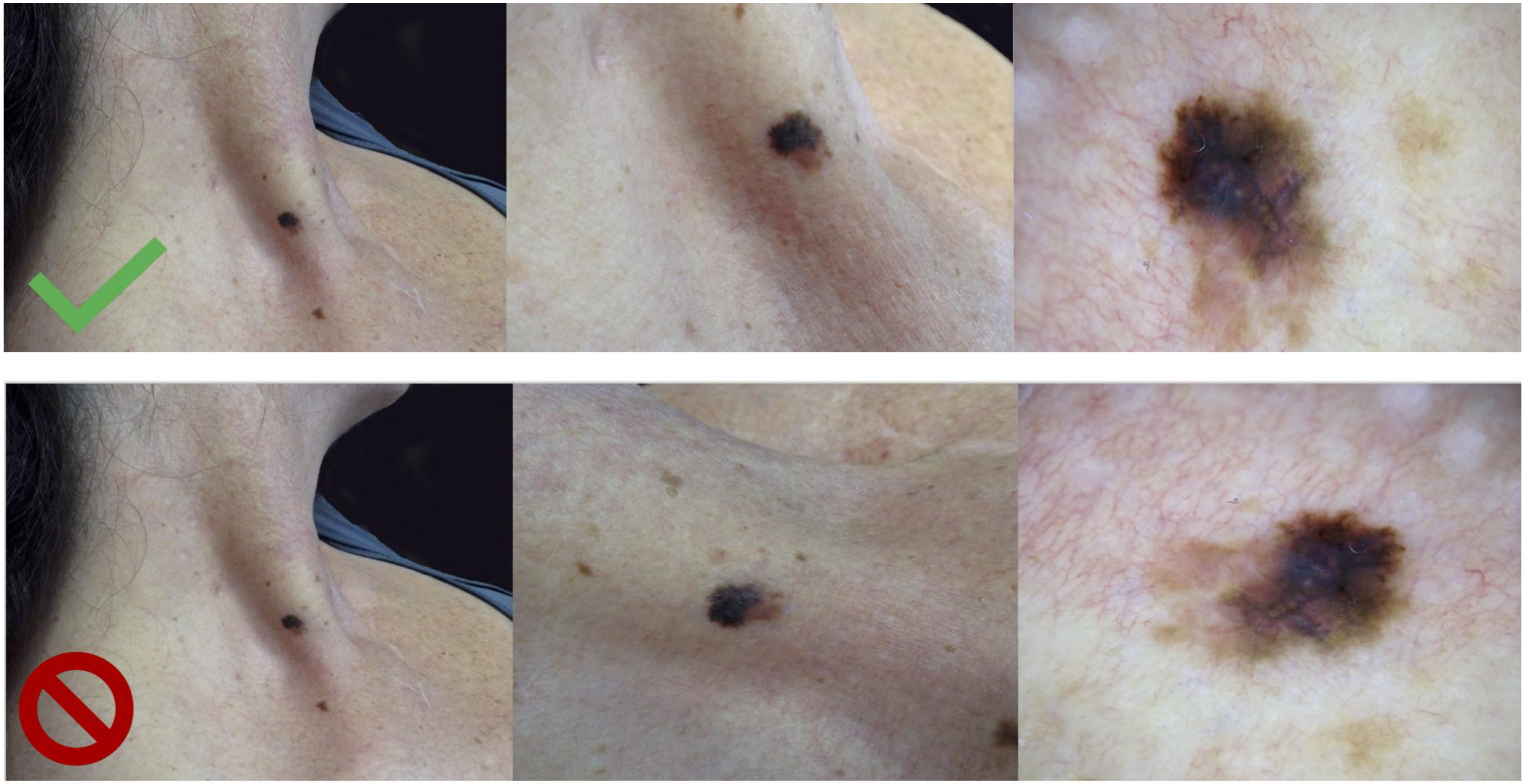

Images courtesy of Clara N. Curiel-Lewandrowski, MD and Delaney B. Stratton, DNP, PhD, University of Arizona / Banner Dermatology Division. These images were developed as part of the International Skin Imaging Collaboration (ISIC) Image Acquisition Guidelines.

Supplemental Figure 2: Checklist for acquisition of clinical images. [32]

Checklist: Acquisition of images for teledermatology

1. LIGHTING
- ☐ Adequate Lighting
- ☐ No shadows or glares
- ☐ Polarized and non-polarized

2. BACKGROUND
- ☐ Solid Color
- ☐ No patterns
- ☐ No jewelry

3. FIELD OF VIEW
- ☐ Balanced & centered
- ☐ Multiple images

4. ORIENTATION
- ☐ Regional
- ☐ Anatomical Identifier
- ☐ Close-up
- ☐ Dermoscopy

5. FOCUS & FIELD
- ☐ Focus on center of lesion
- ☐ Maximum focus possible

6. RESOLUTION
- ☐ Visualize hair follicles
- ☐ Visualize skin markings
- ☐ Visualize regression

6. SCALE
- ☐ Appropriate Scale
- ☐ Distraction-free orientation

Finnane A, et al. Proposed Technical Guidelines for the Acquisition of Clinical Images of Skin-Related Conditions. JAMA Dermatol. 2017;153(5):453-457. doi:10.1001/jamadermatol.2016.6214
